# A neuromechanical computational model of spinal control of locomotion

**DOI:** 10.1186/1471-2202-13-S1-P48

**Published:** 2012-07-16

**Authors:** Sergey N Markin, Alexander N Klishko, Natalia A Shevtsova, Michel A Lemay, Boris I Prilutsky, Ilya A Rybak

**Affiliations:** 1 Department of Neurobiology and Anatomy, Drexel University College of Medicine, Philadelphia, PA, 19129, USA; 2 Center for Human Movement Studies, School of Applied Physiology, Georgia Institute of Technology, Atlanta, GA, 30332, USA

## 

We developed a neuromechanical computational model of cat locomotion that simulated the locomotor movements of cat hindlimbs controlled by spinal locomotor central pattern generators (CPGs, one per limb). In the closed-loop model, CPG operation was adjusted by afferent feedback from the hindlimbs. The CPG model was based on the previous two-level model [4] and included a half-center rhythm generator (RG), producing alternating flexor and extensor activities, and a pattern formation (PF) network operating under control of RG and controlling the synergetic activities of different hindlimb motoneuron pools. This basic model [4] was extended by incorporating additional neural circuits at the PF level allowing the CPG to generate the complex activity patterns of motoneurons controlling two-joint muscles. The model included reflex circuits, mediating reciprocal inhibition between antagonistic motoneurons, recurrent inhibition of motoneurons via Renshaw cells, and di-synaptic excitation of extensors by extensor afferents during stance phase of locomotion.

The hindlimbs with the pelvis and trunk were modeled as a 10 degree-of-freedom sagittal plane system of rigid segments interconnected by frictionless revolute joints. The hindlimb interactions with the ground and other body segments were modeled as linear springs and dampers [1,2]. The two hindlimbs were driven by 18 muscle actuators representing major hindlimb muscles in the cat. The dynamics of each muscle-tendon unit was described by a Hill-type model extended to account for muscle mass, pennation angle, and force-length-velocity properties of muscle and tendon [1,2]. All muscles generated motion-dependent afferent signals which were calculated as functions of muscle length, velocity and force using modified regression equations from [3]. Parameters of the musculoskeletal model were identified by minimizing a mismatch between the computed (based on the recorded hindlimb muscle activity) and experimentally recorded changes in the joint angles, joint moments and ground reaction forces using the simulated annealing optimization algorithm [1,2].

The developed neuromechanical model demonstrated stable locomotion and exhibited realistic patterns of muscle activation, limb kinematics, and ground reaction force dynamics. The model was used for investigation of the role of afferent feedback and the CPG in control of locomotion under different conditions.
